# Clear cell adenocarcinoma of the peritoneum: a case report and literature review

**DOI:** 10.1186/s13048-014-0086-2

**Published:** 2014-10-02

**Authors:** Naoya Shigeta, Kiyoshi Yoshino, Shinya Matsuzaki, Eiichi Morii, Yutaka Ueda, Tadashi Kimura

**Affiliations:** Department of Obstetrics and Gynecology, Osaka University Graduate School of Medicine, 2-2 Yamadaoka, Suita, Osaka 565-0871 Japan; Department of Pathology, Osaka University Graduate School of Medicine, Osaka, Japan

**Keywords:** Clear cell adenocarcinoma, Peritoneum, Endometriosis, Ovarian cancer

## Abstract

Clear cell adenocarcinoma (CCC) is generally thought to originate from ovarian, endometrial, or renal tissue. A CCC of the peritoneum (CCAP) is an extremely rare medical condition and is associated with a poor prognosis. To date, only 10 cases of CCAP have been reported, of which half resulted in death or recurrence within 6 months after initial treatment because CCAP is commonly resistant to multiple drugs. In this report, we present a case of CCAP of the pouch of Douglas coexisting with an endometriosis and we offer a review of the related literature.

## Background

Clear cell adenocarcinoma of the peritoneum is an extremely rare medical condition with poor prognosis. Because of its reality, no standard treatment has been established. Here, we report a case with primary clear cell adenocarcinoma in the pouch of Douglas treated by debulking surgery followed by chemotherapy.

## Case

A 59-year-old postmenopausal woman, gravida 2, para 2, was referred to our hospital for the treatment of a pelvic mass. Her initial complaints were appetite loss and 12 kg weight loss over 3 months. Her past medical history was unremarkable, except for an appendectomy, and her gynecological medical history included administration of a gonadotropin releasing hormone agonist for the treatment of adenomyosis manifesting as severe dysmenorrhea.

**Figure 1:**
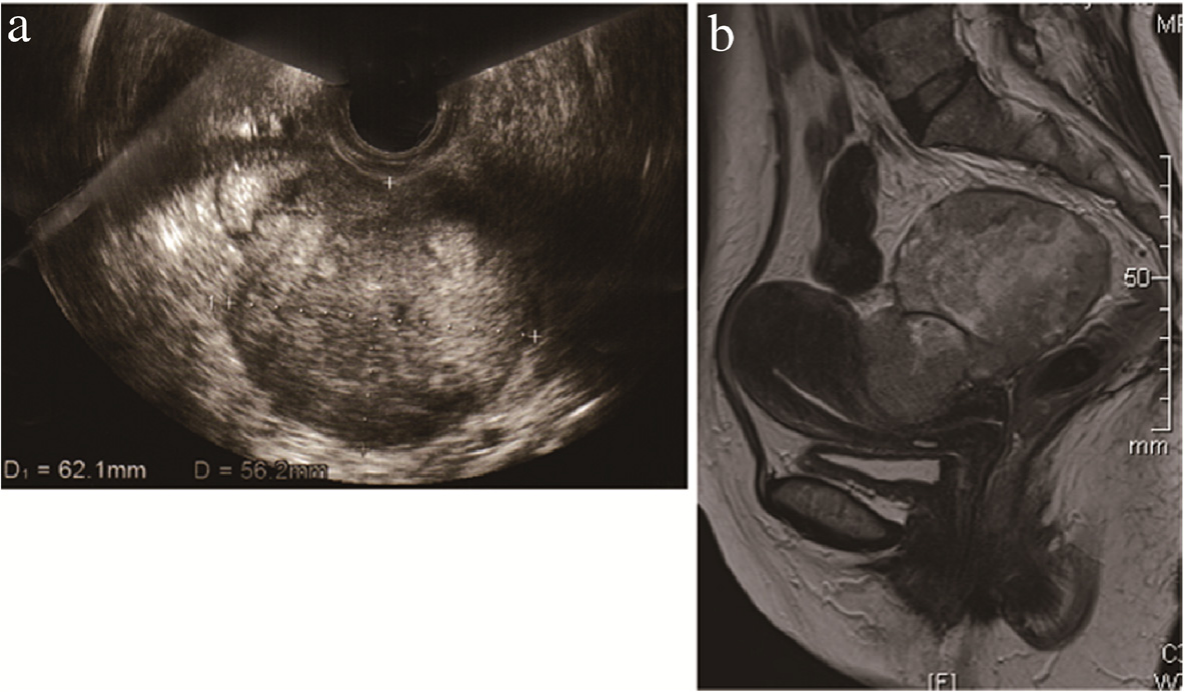
**Clinical imaging of the tumor. a**. Vaginal ultrasonography revealed a 6 cm heterogeneous tumor located in the pouch of Douglas, with a suspected left ovarian involvement. **b**. T2-weighted MRI revealed an 8 cm heterogeneous tumor located at the posterior (intestinal) surface of the uterus, with a suspected rectal invasion.

**Figure 2:**
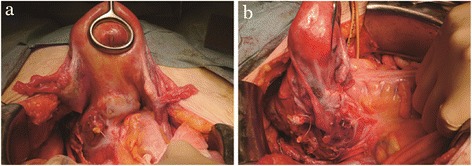
**Image of the operation. a** and **b**. Laparotomy revealed that the tumor did not originate from the ovary, and it was located in the pouch of Douglas. There was a strong adhesion between the tumor and rectum.

On her first visit, a vaginal ultrasound revealed a heterogeneous solid mass with cystic areas which was located approximately 6 cm deep within the pelvis (Figure 1a). Pelvic examination revealed a firm adhesion between the tumor and uterus. Magnetic resonance imaging (MRI) of the pelvis revealed an 8 cm heterogeneous mass located on the posterior (intestinal) surface of the uterus, with possible rectal invasion (Figure 1b). Uterine adenomyosis was also detected by MRI. Fluorodeoxyglucose (FDG) positron emission tomography–computed tomography revealed a mass on the posterior surface of uterus with FDG uptake in the nodules adjacent to right side of the mass and in the pelvic and obturator lymph nodes. The serum level of cancer antigen (CA) 125 was slightly elevated to 76 U/mL (normal limit: 35 U/mL), whereas CA19-9 and carcinoembryonic antigen (CEA) levels were within normal ranges. Taken together, we made a preoperative diagnosis of a left ovarian malignant tumor. During surgery, surprisingly, the tumor was located in the pouch of Douglas and was found to not originate from the ovary (Figure 2a and b). The tumor was adherent to the uterus at the left uterosacral ligament and had also invaded the rectum. Both ovaries appeared normal. During surgery, the tumor capsule was ruptured; because of this, the tumor was removed together with the rectum, uterus and both ovaries. Total abdominal hysterectomy, bilateral salpingo-oophorectomy, partial infracolic omentectomy, pelvic lymphadenectomy, and low anterior resection of the rectum were performed. There were no macroscopic residual tumors, thus the procedures performed were considered an optimal surgical treatment.

**Figure 3:**
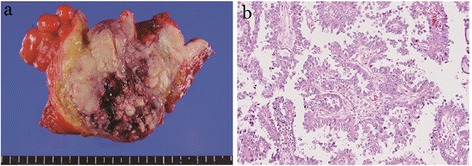
**Gross and microscopic appearance of the tumor. a**. A photograph of the cut surface of the tumor. A multilobular cyst with a yellowish necrotic solid part was observed. Both ovaries were normal. **b**. Histopathological features of the tumor. Clear cells lined the glandular and papillary structure. A hobnail arrangement of the cells was seen (hematoxylin and eosin; original magnification: ×400).

On macroscopic evaluation, a multilobular cyst with a yellowish necrotic solid part was found on the cut surface of the tumor (Figure 3a). The tumor had invaded the uterine cervix and rectum, up to the level of the mucosa. Both ovaries were visually tumor free. Microscopic examination of the tumor revealed a clear cell adenocarcinoma. Glandular and papillary structures were lined by clear cells and a hobnail arrangement of cells within the glands was found (Figure 3b). The clear cells contained eosinophilic cytoplasm. Immunochemical staining for cytokeratin 7 was positive, whereas staining for cytokeratin 20, caudal-type homeobox protein 2, and estrogen receptor were negative. These results indicated that the tumor did not originate from the rectum. Microscopically, tumor cells were not observed in either ovary. Lymphatic vessel invasion was observed in the invasive area of the rectum, and 3 of 3 resected pararectal lymph nodes (#251) were involved by the tumor. On the other hand, there were no metastases in 28 of resected regional pelvic lymph nodes. An adenomyoma was found in the uterus and endometriotic lesions were found at the peritoneum near the tumor. Thus, the tumor was diagnosed as CCAP with endometriosis. Postoperative staging was FIGO stage IIB (pT2bN0M0).

Six courses of combination chemotherapy with paclitaxel and carboplatin (TC) as adjuvant therapy was planned. At 3 months after the primary surgery, the patient presented with strangulated ileus, which was corrected by a second surgery. No recurrent disease was observed in the abdomen at the surgery. Then, the patient completed the planned adjuvant therapy and has remained well with no evidence of disease 10 months after the primary surgery.

## Discussion

Primary peritoneal carcinoma was first described by Swerdlow in 1959 [1]. The Gynecologic Oncology group defines the diagnostic criteria for primary peritoneal carcinoma, where both ovaries are of normal size, with an extraovarian involvement greater than the surface ovarian involvement [2]. Our case met this criterion and was histologically diagnosed as CCC.

To investigate reports of the incidence of CCAP, we performed a search of the PubMed database using the key words “clear cell” or “peritoneal” and “primary”. The search was limited to the English literature and CCAP cases. We identified 10 cases that are summarized in Table 1 [3–12]. These limited cases were reviewed to provide characteristics of CCAP and identify potentially effective treatments. As summarized in Table 1, 10 of the 11 cases (including our case) were initially treated by debulking surgery. Complete resection was performed in six cases and the residual tumor size was >2 cm in four cases. Eight cases received chemotherapy (seven postoperatively and one with primary chemotherapy). The chemotherapy regimens performed were TC [6,12], irinotecan hydrochloride + cisplatin (CPT-P) [9], and cyclophosphamide + carboplatin + cisplatin (CCP) [6].

**Table 1:** **Summary of clear cell adenocarcinoma of the peritoneum**

**Case**	**Authors**	**Age**	**Tumor size and location**	**Past history or concurrent disease**	**Concurrent endometriosis**	**Treatment**	**Residual tumour**	**Outcome**
1	Evans et al. [3]	54	18×13 cm	Both Ov EM, AM, EH	Yes	DS followed by RT (pelvic; 4500R,upper abdomen;3000 R)	None	NA
			sigmoid mesocolon					
2	Lee et al. [4]	67	6 cm	Concurrent EMA G1 of Ut (stageIb)	No	DS+TAH+BSO	>2 cm	NA
			Pelvic					
3	Tziortzioti et al. [5]	62	0.5-2 cm	Concurrent CCC in EP	No	DS+TAH+BSO +OM followed by CHT (6 cycles, regimen NA)	>2 cm	DOD at 6 mo
			Peritoneal and omental					
4	Ichimura et al. [6]	45	NA	Both Ov EM	Yes	DS+TAH+BSO followed by CHT (3 cycles of CPA,CDDP, CBDCA)	None	ROD at 32 mo
			Pelvic					
5	Hama et al. [7]	53	NA	EM	Yes	DS+BSO followed by CHT (regimen NA)	>2 cm	DOD at 5 mo
			Ascites and small peritoneal solid lesions					
6	Terada et al. [8]	49	3 cm, 2 cm	Past EMA G3 of Ut (TAH+BSO)	No	DS	None	NED at 6 mo
			Gastric peritoneal, splenic hilus					
7	Takano et al. [9]	53	5 cm	None	No	DS followed by CHT (1 cycle of CPT-11,CDDP, 1 cycle of TC)	>2 cm	DOD at 5 mo
			Upper abdomen between liver and diaphragm and omentum					
8	Takano et al. [9]	66	15×20 cm	None	No	DS+TAH+BSO+OM+PLD+PAD followed by CHT (6 cycles of CPT-11, CDDP)	None	NED at 20 mo
			Infracolic omentum and peritoneum of right abdominal wall					
9	Matsuo et al. [10]	37	14×13 cm	EM	Yes	DS followed by CHT (6 cycles of TC), Secondary devulking surgery	None	ROD at 18 mo
			Abdominal scar of EM surgery					
10	Muezzinoglu et al. [11]	54	25 cm	Peritoneal EM	Yes	DS+TAH+BSO following by CHT (regimen NA)	None	NED at 12 mo
			Abdominal					
11	Johnson et al. [12]	54	5.6×3.7×3.5 cm	3xMMs, TAH+BSO for LM and menorrhagia	No	CHT (6 cycles of TC) followed by EBR followed by the interstitial HDR BT boost	<2 cm	ROD at 4 mo
			proximal vagina and vaginal cuff					
12	This report	59	7 cm	AM, EM	Yes	DS+TAH+BSO+PLD followed by CHT (6 cycles of TC)	None	NED at 5 mo
			Pelvic (pouch of douglas)					

EM, endometriosis; Ov, ovary; Ut, uterus; AM, adenomyosis); EH, endometrial hyperplasia; EP, endometrial polyp; LM, leiomyoma; EMA, endometrial adenocarcinoma; CCC, clear cell adenocarcinoma, MM, myomectomy; DS, debulking surgery; PLD, pelvic lymph node dissection; PAD, para-aortic lymph node dissection; OM, omentectomy; CHT, chemotherapy; RT, radiotherapy; EBR, external beam radiotherapy; BT, brachytherapy; CPT-11, irinotecan hydrochloride; CDD, cisplatin; CBDCA, carboplatin; CPA, cyclophosphamide; NED, no evidence of disease; ROD, recurrence of disease; DOD, dead of disease; NA, not available; mo, months.

We offered TC combination chemotherapy to our patient. One report had found that a TC combination therapy was effective for the treatment of CCAP. Outcomes were poor in five cases. Of these, three patients died within 6 months, and disease recurrence had occurred in two cases at 4 months and 32 months, respectively, after the initial therapy [6,8–11]. Three patients died within 6 months and each had a residual tumor of >2 cm [5,7,9]. Early recurrence occurred in the inoperable patients. The characteristic features of the cases with particularly poor prognosis were large residual tumors after primary surgery and inoperability. On the other hand, there were three cases with relatively good prognosis [8,9,11]. The primary features of these cases were having had an optimal or complete debulking surgery followed by chemotherapy.

CCAP can closely resemble ovarian clear cell adenocarcinoma (OCC) and it is difficult to distinguish CCAP from OCC by preoperative imaging alone, especially if the tumor is located in the pelvis close to the ovary or uterus, even though the radiological features of CCAP have been reported in some cases. One report cited a multicystic mass with a multiseptate appearance and a heterogeneous solid part arising from the peritoneum, suggesting the occurrence of a CCAP [12].

In our case, we did not observe such features and we were unable to determine whether the pelvic tumor originated from the peritoneum. We recommend further imaging studies to establish a definitive diagnosis of CCAP. An endometriosis coexisted with the CCAP in our case. CCC is seen most frequently in the ovary and is often associated with pelvic endometriosis [6], as an association with endometriosis has been well established [9]. CCC occasionally develops from extraovarian endometriosis [9,13]. There are several reports of CCAP cases with coexisting endometrial lesions. In some reports, endometriosis was a required precursor of CCC in peritoneal locations. However, others found that the tumor arose de novo from the peritoneum [4]. The pathogenesis of carcinoma coexisting with endometriosis has not been fully elucidated. In past reports, two possible theories for the association of carcinoma and endometriosis were mentioned. These theories included a genetic defect in already existing endometriosis or a defect in the immune response of patients with endometriosis that leads to the progression of endometriosis to subsequent malignant transformation [2]. Hence, the pathogenesis of CCAP associated with endometriosis remains controversial.

The pathogenesis of peritoneal serous carcinoma is similar to that of ovarian cancer and may be dependent on its origin from ovarian tissue remnants in the peritoneum remaining from embryonic development or from the mesoderm that gives rise to both the ovarian epithelium and peritoneum [14]. Therefore, female pelvic and abdominal mesothelium may give rise to primary peritoneal carcinoma resembling ovarian cancer [5]. In addition to these reports, non-serous carcinoma of the peritoneum has also been reported [15]. Therefore, CCAP may arise de novo from the peritoneum.

## Conclusions

Here, we report a case of primary clear cell adenocarcinoma arising from the pouch of Douglas. Although 10 similar cases have been reported, there is as yet no established treatment regimen for this disease. Combination chemotherapy with debulking surgery has been suggested for the treatment of residual tumors <2 cm with postoperative chemotherapy, such as TC or CPT-P. To establish an effective treatment regimen for CCAP, a review of a larger number of cases is still needed.

## Consent

Written informed consent was obtained from the patient for publication of this Case report and any accompanying images. A copy of the written consent is available for review by the Editor-in-Chief of this journal.
